# Modulation of the Tomato Fruit Metabolome by LED Light

**DOI:** 10.3390/metabo10060266

**Published:** 2020-06-26

**Authors:** Nikolaos Ntagkas, Ric C. H. de Vos, Ernst J. Woltering, Celine C. S. Nicole, Caroline Labrie, Leo F. M. Marcelis

**Affiliations:** 1 Horticulture and Product Physiology, Wageningen University and Research, 6700 AA Wageningen, The Netherlands; ernst.woltering@wur.nl (E.J.W.); leo.marcelis@wur.nl (L.F.M.M.); 2 Bioscience, Wageningen University and Research, 6700 AA Wageningen, The Netherlands; ric.devos@wur.nl; 3 Food and Biobased Research, Wageningen University and Research, 6700 AA Wageningen, The Netherlands; 4 Signify Research, 5656 AE Eindhoven, The Netherlands; celine.nicole@signify.com; 5 Greenhouse Horticulture, Wageningen University and Research, 2665 ZG Bleiswijk, The Netherlands; caroline.labrie@wur.nl

**Keywords:** tomato, metabolomics, light regulation, irradiance, light spectrum

## Abstract

Metabolic profiles of tomatoes change during ripening and light can modulate the activity of relevant biochemical pathways. We investigated the effects of light directly supplied to the fruits on the metabolome of the fruit pericarp during ripening. Mature green tomatoes were exposed to well-controlled conditions with light as the only varying factor; control fruits were kept in darkness. In experiment 1 the fruits were exposed to either white light or darkness for 15 days. In experiment 2, fruits were exposed to different light spectra (blue, green, red, far-red, white) added to white background light for seven days. Changes in the global metabolome of the fruit pericarp were monitored using LCMS and GCMS (554 compounds in total). Health-beneficial compounds (carotenoids, flavonoids, tocopherols and phenolic acids) accumulated faster under white light compared to darkness, while alkaloids and chlorophylls decreased faster. Light also changed the levels of taste-related metabolites including glutamate and malate. The light spectrum treatments indicated that the addition of blue light was the most effective treatment in altering the fruit metabolome. We conclude that light during ripening of tomatoes can have various effects on the metabolome and may help with shaping the levels of key compounds involved in various fruit quality characteristics.

## 1. Introduction

Tomato is an important crop widely produced around the world. Due to its nutritional content, tomato plays an important role in human health [1]. An adequate understanding of the environmental factors and the underlying physiological processes that regulate the nutritional content of the tomato fruits is essential for the production of fruits with enhanced nutritional value. Furthermore, due to its well-studied genome and suitability for genetic manipulation, the tomato is a model crop for the study of a variety of metabolic processes [2].

Tomato fruits contain a wide range of metabolites, with some of the most important being carotenoids; flavonoids and other phenolic compounds; and taste and aroma-related compounds. Tomato contains considerable amounts of metabolites that act as antioxidants (phenolic compounds and carotenoids) and vitamins (e.g., vitamins C and E). It is most known as a crop rich in the carotenoid all-trans lycopene. Consumption of foods rich in lycopene has been proposed to be related to the prevention of multiple types of cancer [1,3] and cardiovascular diseases [4]. Certain claimed health benefits of lycopene such as prostate cancer prevention remain debatable to date [5,6]. Apart from all-trans lycopene, tomatoes contain carotenes which contribute to the specific color of orange-colored varieties and are also known to have beneficial effects on the human body by acting as antioxidants and being the main vitamin A precursors [7]. Furthermore, carotenoids are the precursors of a variety of volatiles in fruits, generated through the action of carotenoid-cleavage enzymes [8].

The tomato is a climacteric fruit that is able to ripen after harvest. The climacteric peak in internal ethylene concentration, typically observed during the color transition from green to red, stimulates a variety of developmental processes resulting in drastic changes in the metabolome. When the fruits enter the breaker stage, chlorophylls are broken down while flavonoids and carotenoids increase dramatically [9]. Besides the visible pigment changes, other phenomena also occur during ripening of the tomato fruits. Typically, starch is broken down to hexoses and the production of aroma volatiles is upregulated. Furthermore, modifications of the cell walls occur, resulting in the loss of firmness [10,11,12]. These ripening-related changes determine to a large extent the typical sensory characteristics of tomatoes.

Light is the primary source of energy in plants and as such it affects the metabolome not only of the leaves but also of the fruits. Besides being present in leaves, phytochromes [13] and cryptochromes [14] have been found in the pericarps of tomato fruits. Therefore, not only the amount but also the spectrum of the received light may potentially influence the metabolome of tomato. Higher irradiance levels on tomato fruits can stimulate, for instance, the accumulation of ascorbic acid in the pericarp, resulting in a 5-fold increase [15,16] through an overall increased photosynthetic activity in the fruit [17,18]. The spectrum had only small effects on this vitamin [16]. The light spectrum may also influence other fruit metabolic pathways, such as those involved in pigmentation. For instance, in cranberries the application of red light increased the anthocyanin content as compared to white and far-red light [19]. To better characterize how light regulates the quality and shelf life of fruits, research may be expanded to both nutritional health-related and taste and aroma-related metabolites. This knowledge becomes highly relevant with the increasing integration of LEDs in horticultural lighting systems [20,21]. Use of LEDs potentially opens up a wide range of applications, including the regulation and fine-tuning of the crop metabolome. LEDs have not only been related to a high energy efficiency, but they also allow application of light with specific wavelengths [22] in order to investigate light spectrum-dependency of plant metabolic pathways and the potency to fine-tuning specific crop quality characteristics related to metabolite composition.

The abiotic environment, especially light, during the preharvest and postharvest parts of the tomato supply chain plays an important role in the fruit ripening process, as it may affect the metabolomes of the fruits. The aim of this research was to study the effect of light on the metabolic profiles of tomato fruits during their ripening. We tested the hypothesis that both light quantity (increased duration of white light supply) and light quality (its spectrum) can directly modulate the fruit composition, as previously observed for ascorbic acid [17,18]. Detached tomato fruits were exposed to treatments with and without white LED light, and to different light spectra by additional illumination using monochromatic LEDs. Both targeted and untargeted metabolomics analyses of fruit pericarp were performed, which provided us the ability to study multiple changes in the metabolome simultaneously. We discuss the observed changes in metabolite profiles in relation to the potential impact on visual quality (pigment), nutritional quality (several vitamins and presumed health-related compounds) and organoleptic characteristics (flavor-related compounds, sugars, acids).

## 2. Results

### 2.1. Targeted and Untargeted Metabolomics

For each metabolomics profiling platform the analyses were performed on all samples from both the irradiance and light quality experiments simultaneously and in a randomized order. Per platform, five or seven quality control samples (QCs), consisting of repetitive extractions from a pooled sample of all fruit powders, were simultaneously prepared and equally distributed between the real samples, in order to estimate the overall analytical variation per compound. The targeted analysis of lipid-soluble isoprenoids by HPLC-PDA-Fl resulted in absolute levels of nine well-known lipid-soluble tomato isoprenoids. Based on the QCs (*n* = 7), the analytical variation (standard error of mean) for these nine isoprenoids was on average 12.6%, ranging from 4.7% for alpha-tocopherol to 25.9% for zeaxanthin. The untargeted LCMS platform yielded relative abundance levels of 437 mainly semi-polar metabolites, of which 108 were putatively identified; for these LCMS compounds the average analytical variation, for 340 out of the 437 compounds present in all five QCs, was 24.6% (Table S1). Finally, the untargeted GCMS platform yielded relative levels of 108 polar compounds, of which 22 were putatively identified; in this GCMS analyses the average analytical variation over all compounds was 38% (Table S2).

### 2.2. Time Course of Metabolite Profiles in Light Compared to Darkness (Experiment 1)

As was judged by the color and firmness changes [16], the ripening process of the detached tomato fruits proceeded more or less similarly in both the light (500 μmol m^−2^ s^−1^) and dark treatments. In both treatments, the visual assessment of fruit color indicated that the green color had disappeared by day 9, at which time the fruits entered their breaker stage (transitionary stage between green and red coloration). From day 9 onwards, the color of both light and dark-exposed fruits gradually developed further, though slightly slower in in the dark-ripened fruits [16].

**Figure 1:**
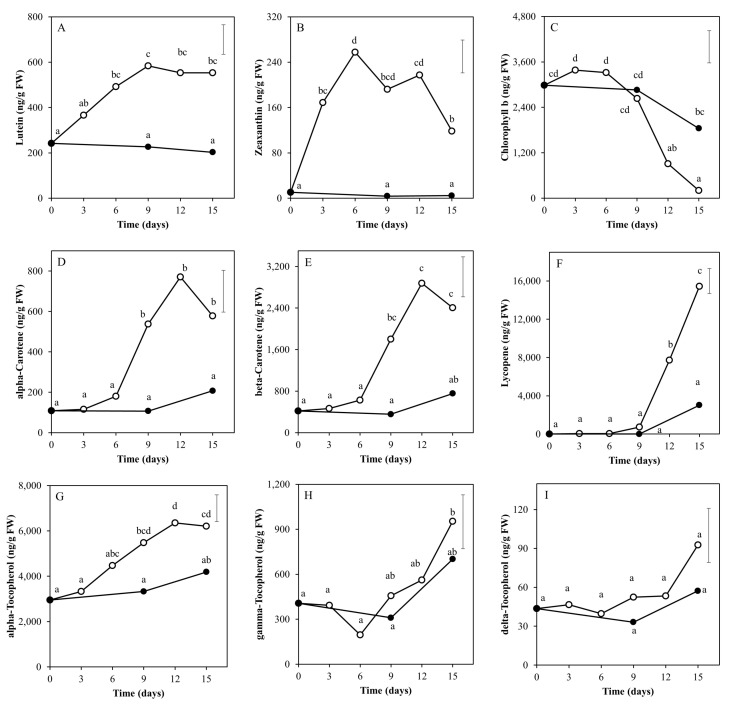
Levels of lipid-soluble metabolites (isoprenoids) in tomato pericarp from experiment 1, measured by LC-PDA-Fl. The fruits were treated with either white light (500 μmol m^−2^ s^−1^; open symbols) or darkness (closed symbols) for 15 days and sampled at indicated days. Error bars indicate the least significant difference; letters (**A**–**I**) indicate significant differences between treatments or days at α = 0.01, *n* = 3 (3 pools of 2 fruits each).

The targeted HPLC analyses of the lipid-soluble isoprenoids (Figure 1) indicated that the chlorophyll b content (Figure 1C) significantly declined after nine days and that this decline was faster in light than in darkness. In the light, all carotenoids and tocopherols, except the relatively low abundant delta-tocopherol (Figure 1I), showed an increase during the first 3–12 days, some after an initial lag phase. These figures suggest that after 12 days the levels of lutein, alpha-carotene, beta-carotene and alpha-tocopherol (Figure 1A,D,E,G) were more or less stabilized; zeaxanthin decreased again (Figure 1B); and lycopene, gamma-tocopherol and possibly delta-tocopherol still increased (Figure 1F,H,I). The time courses for lutein, alpha-tocopherol and alpha and beta-carotene might be considered to be similar but with minor shifts in time. In fruits placed in darkness, there were no significant changes over time for these isoprenoids analyzed, except for chlorophyll b (Figure 1C). However, the fact that at day 15 alpha-carotene, beta-carotene, lycopene and all three tocopherols showed the tendency to be increased as compared to day 0 and day 9, although not significantly at α = 0.01, suggests that the time-related increase in these compounds is delayed in fruits kept in darkness as compared to their exposure to white LED light.

**Figure 2:**
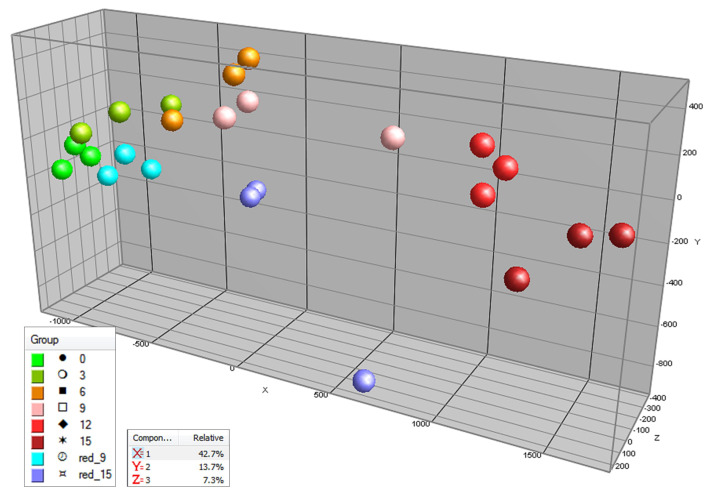
Principal component analysis (PCA) of tomato samples from experiment 1, based on their variations in relative abundance of 437 mainly secondary metabolites detected by LCMS based untargeted metabolomics. The fruits were treated with either light (500 μmol m^−2^ s^−1^) or darkness for 15 days. Samples of the light treatment were measured every three days and for the dark treatment at 9 and 15 days after the beginning of the experiment (t = 0 days). PC1 = *X*-axis = 42.7% explained variation; PC2 = *Y*-axis = 13.7% explained variation; PC3 = *Z*-axis = 7.3% explained variation. (Light treatment: t = 0 days—light green points, t = 3 days—dark green points, t = 6 days—orange points, t = 9 days—pink points, t = 12 days—light red points, t = 15 days—dark red points. Dark treatment: t = 0 days—light green points, t = 9 days—light blue points, t = 15 days—purple points.)

In order to determine the overall effect of light or darkness on the tomato pericarp metabolites detected by the untargeted LCMS approach, which mainly detects semi-polar secondary metabolites, a Principal Component Analysis (PCA) was carried out based on the variation in the relative intensity values of all 437 compounds detected (Figure 2). The main effect observed (first principle component-PC, X-axis) obviously corresponds to the time of treatment; i.e., fruit ripening. At both day 9 and day 15, the light-treated fruits are on average more separated from day 0 (mature green fruits) then the fruits that were placed in darkness, suggesting faster fruit ripening. The second PC (*Y*-axis) coincides with the contrast of light versus dark treatments, suggesting a differential regulation of fruit biochemical pathways during ripening in light versus darkness. At day 15 of treatment, the relative levels of 79 compounds, i.e., 18% of all LCMS compounds detected, were significantly (*p* < 0.01, *t*-test, *n* = 3) higher in light compared to darkness, while only four metabolites i.e., 0.9%, were lower (Table S3).

**Figure 3:**
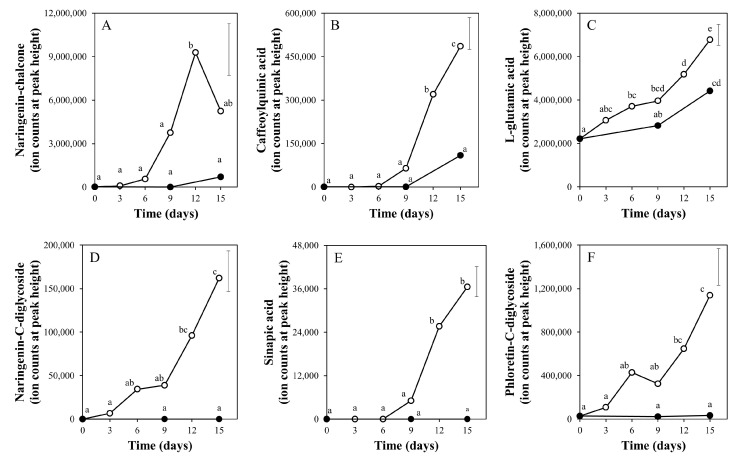
Relative levels of selected metabolites in tomato pericarp from experiment 1, measured by LCMS. The fruits were treated with white light (500 μmol m^−2^ s^−1^; open symbols) or darkness (closed symbols) for 15 days and sampled at indicated days. Error bars indicate the least significant difference; letters (**A**–**F**) indicate significant differences between treatments or days at α = 0.01, *n* = 3 (three pools of two fruits each).

We subsequently selected a number of compounds for further manual identification, based on their observed accurate masses and comparison with tomato fruit metabolite databases, and for investigating their specific abundance patterns upon treatment with light and darkness (Figure 3). Presented metabolites were interesting from a metabolism stand-point. The phenolic compounds naringenin-chalcone, naringenin-C-diglycoside, sinapic acid, caffeoylquinic acid and phloretin-C-diglycoside, and the umami-taste related amino acid L-glutamic acid, markedly increased with time of treatment, and in the end (day 15) were significantly more abundant in light-exposed fruits than in fruits kept in darkness (Figure 2), with naringenin-C-diglycoside and sinapic acid being undetectable in dark-treated fruits at all three time points analyzed. A variety of other naringenin and phenylpropanoids, and l-threonate and the flavonol-glycoside rutin, were also higher in fruits exposed to light compared to darkness (Table S3). In light, naringenin-chalcone increased up to 12 days and then decreased again. In the light, in total 156 (35.7%) of all LCMS compounds detected were significantly increased at day 15 of experiment as compared to the initial mature green fruits; meanwhile, 31 compounds (6.8%) decreased (Table S4). For fruits stored in darkness, only 31 metabolites were significantly changed between day 15 and day 0, of which 20 were also changed in the light treatment, thereby suggesting not only a faster but also a differential regulation of metabolism in fruits ripening under light.

**Figure 4:**
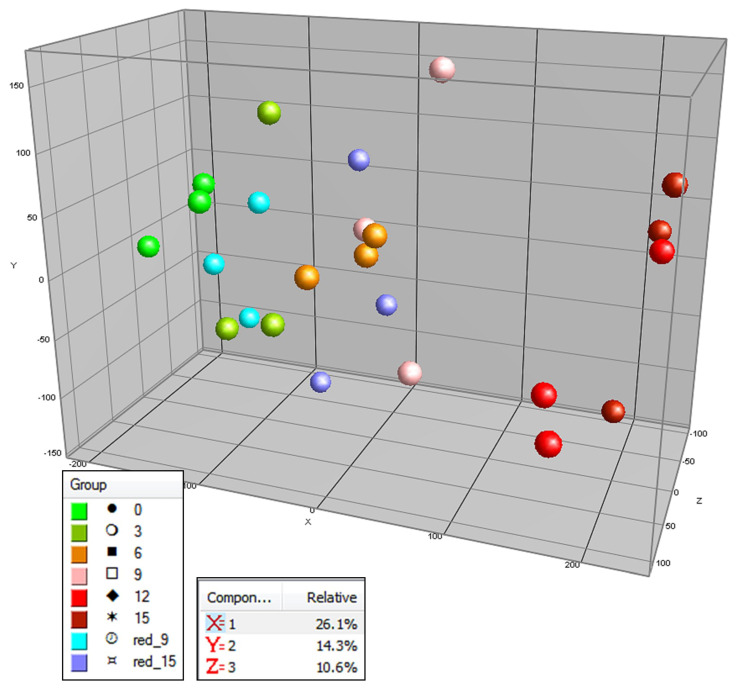
Principal component analysis (PCA) of tomato samples from experiment 1, based on their variations in relative abundance of 108 mainly primary metabolites detected by GCMS based untargeted metabolomics. The fruits were treated with either light (500 μmol m^−2^ s^−1^) or darkness for 15 days. Samples of the light treatment were measured every three days and for the dark treatment at 9 and 15 days after the beginning of the experiment (t = 0 days). PC1 = *X*-axis = 26.1% explained variation; PC2 = *Y*-axis = 14.3% explained variation; PC3 = *Z*-axis = 10.6% explained variation. (Light treatment: t = 0 days—light green points, t = 3 days—dark green points, t = 6 days—orange points, t = 9 days—pink points, t = 12 days—light red points, t = 15 days—dark red points. Dark treatment: t = 0 days—light green points, t = 9 days—light blue points, t = 15 days—purple points.)

Next to influencing the global composition of secondary metabolites, the light exposure also exerted a clear effect on 108 primary metabolites, as detected by GCMS (Figure 4). Again, the main effect (first PC; *X*-axis) coincided with time of treatment (fruit ripening time), with light-treated fruits showing a faster ripening compared to the dark-treated ones. In contrast to the PCA plot based on secondary metabolites (Figure 2), with the primary metabolites we did not observe a separate grouping of dark versus light-treated fruits in the PCA plot (Figure 4).

**Figure 5:**
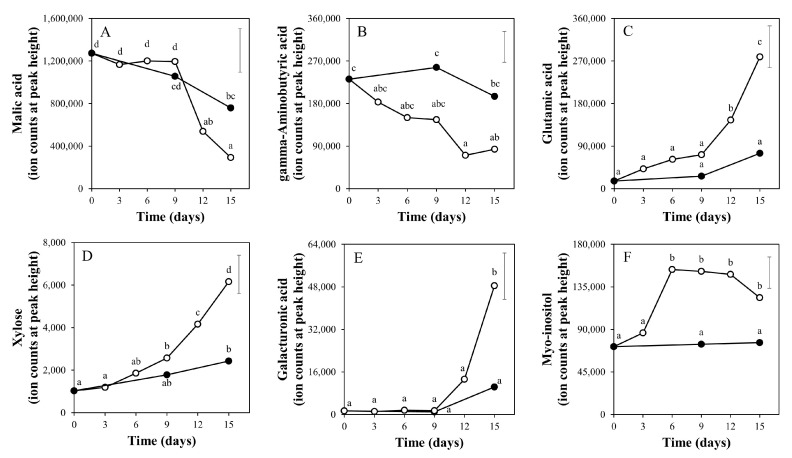
Relative levels of selected metabolites in tomato pericarp from experiment 1, measured by GC-MS. The fruits were treated with white light (500 μmol m^−2^ s^−1^; open symbols) or darkness (closed symbols) for 15 days and sampled at indicated days. Error bars indicate the least significant difference; letters (**A**–**F**) indicate significant differences between treatments or days at α = 0.01, *n* = 3 (three pools of two fruits each).

Figure 5 shows some selected differentially accumulating primary metabolites. The presented metabolites were interesting from a metabolism stand-point. Glutamic acid, xylose, galacturonic acid and myo-inositol [18] gradually increased over time, specifically in the light, and in the end were significantly higher in the light than in the dark (Figure 5C to Figure 5F). It is worth noting that the observed patterns of fruit glutamic acid levels in light and dark treatments, as determined by this GCMS platform (Figure 5C), are very similar to the patterns detected by the LCMS platform (Figure 3C), thereby showing the robustness of the applied metabolomics approaches. Both malic acid and gamma-aminobutyric acid (GABA) decreased over time and at day 15 malic acid were 2.6-fold lower in the light-treated fruits (Figure 5A); meanwhile, GABA was significantly lower at 12 days with no significant difference at 15 days (Figure 5B). At the end of the treatments the levels of 19 compounds (17.4% of total GCMS metabolites) were significantly higher, while only 2 (1.8%) were lower in light compared to the dark treatment (Table S5). In total, 16 (14.7%) GCMS compounds were increased and only six (5.5%) were decreased after the 15 days of light exposure as compared to the initial mature green fruits (Table S6). Twelve (11%) GCMS compounds were significantly affected by 15 days of storage in darkness.

### 2.3. Effect of Light Spectrum (Experiment 2)

**Figure 6:**
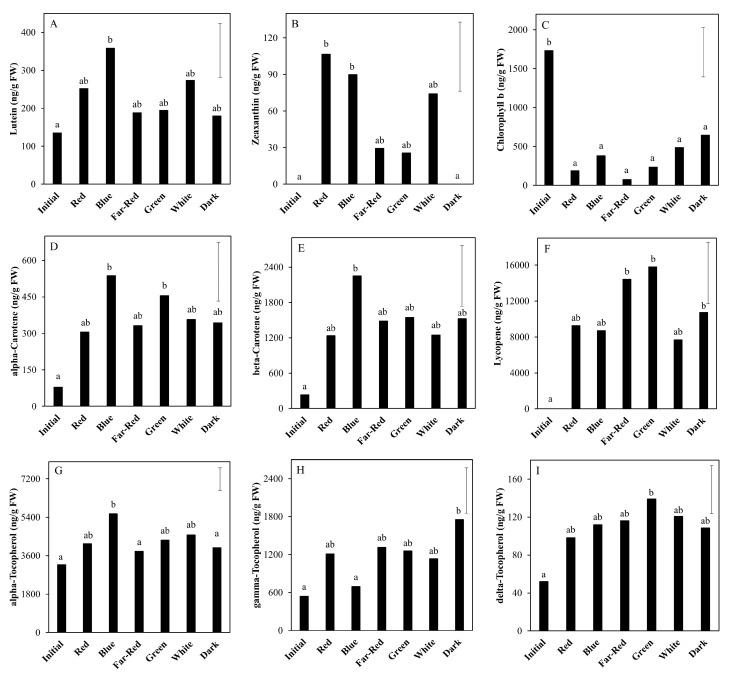
Levels of lipid-soluble metabolites (isoprenoids) in tomato pericarp from experiment 2, measured by LC-PDA-Fl. The fruits were exposed to white light (background) supplemented with monochromatic red, blue, far-red and green light, and to broad-spectrum white light (white) or no light at all (dark) for seven days. Initial values represent fruits at start of experiment. Error bars indicate the least significant difference; letters (**A**–**I**) indicate significant differences between light treatments at α = 0.01, *n* = 3 (three pools of two fruits each).

We subsequently investigated the effect of light spectral compositions on the tomato metabolome, as compared to white light (see above) and no light at all (darkness). Here the fruits were exposed to different LED light spectra, in addition to a background of white light, for seven days. In the group of carotenoids, lutein (Figure 6A) was only significantly increased by the blue light treatment, while zeaxanthin (Figure 6B) was increased by all light quality treatments but most specifically in the red and blue light (this compound was undetectable in initial fruits and dark-treated fruits). Alpha-carotene increased in both blue and green light (Figure 6D), while beta-carotene increased only in blue light (Figure 6E). The three tocopherols (Figure 6G–I) also showed variable responses to the light spectrum treatments: alpha-tocopherol was positively affected only by addition of blue light, gamma-tocopherol was highest in darkness and delta-tocopherol only increased significantly in fruits under green light, as compared to the initial green fruits. Chlorophyll (Figure 5C) decreased to more or less similar levels by all treatments without a particular wavelength effect. Lycopene levels (Figure 6F) increased more in far-red and green light as compared to the white, red or blue LED addition (Figure 6F).

**Figure 7:**
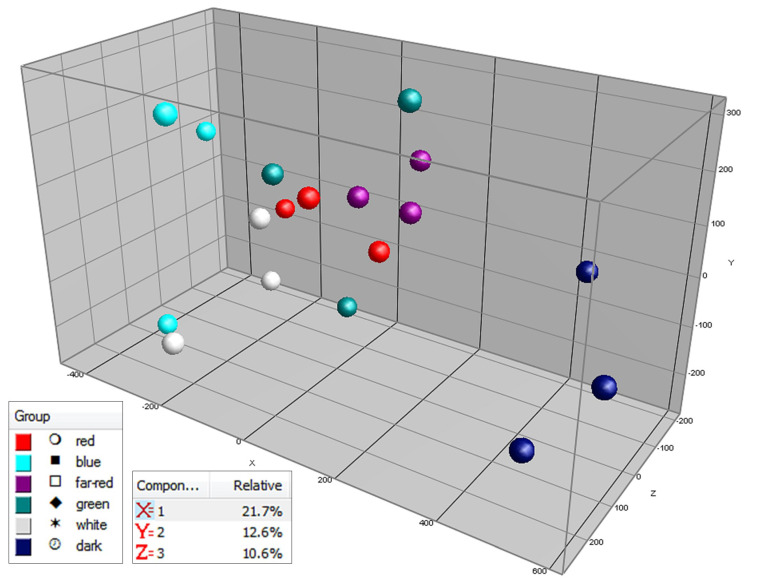
Principle component analysis (PCA) of tomatoes treated with different light qualities, based on 437 tomato pericarp metabolites detected by LC-MS based untargeted metabolomics. The fruits were treated with different light spectrum treatments (350 μmol m^−2^ s^−1^) and darkness for seven days (experiment 2). PC1 = *X*-axis = 21.7% explained variation; PC2 = *Y*-axis = 12.6% explained variation; PC3 = *Z*-axis = 10.6% explained variation. (Red treatment—red points, blue treatment—light blue points, far-red treatment—purple points, green treatment—dark green points, white treatment—white points, dark treatment—dark blue points.)

In order to establish the effects of these contrasting light spectra on semi-polar and polar metabolites, the same fruits were also analyzed using untargeted LCMS and GCMS-based metabolomics approaches, respectively. A PCA based on the 437 LCMS data of all samples, including the fruits at the start of experiment (Figure S1) clearly indicates that the first PC corresponds to differences between day 0 and day 7 of experiment; i.e., ripening time effect (cf. Figure 2 and Figure 4). Thus, in order to zoom in on possible light quality effects, we performed a so-called local PCA based on the seven day-treated fruits only (Figure 7). In this PCA, all light-treated fruit samples were clearly separated from the fruits kept in darkness. Blue light treatment exerted the largest effect (largest distance to the dark-treated fruits on PC1) followed by white light; far-red, green and red light treatments exerted intermediate effects.

**Figure 8:**
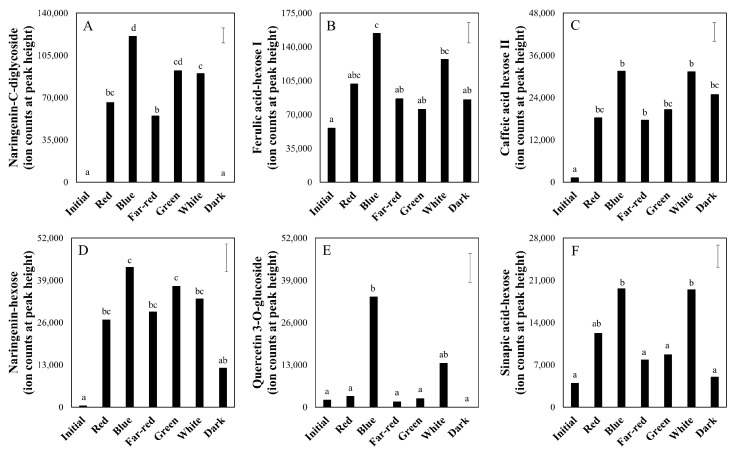
Relative levels of selected metabolites in tomato pericarp from experiment 2, measured by LC-MS. The fruits were treated with white light (background) supplemented with monochromatic red, blue, far-red and green light, and broad-spectrum white light (white) or no light at all (dark) for seven days. Error bars indicate the least significant difference; letters (**A**–**F**) indicate significant differences between light treatments at α = 0.01, *n* = 3 (three pools of two fruits each).

From these compounds detected by LCMS, we again selected some secondary metabolites to show their individual responses to the various seven-day light quality treatments (Figure 8). The presented metabolites were interesting from a metabolism stand-point. The relative levels of naringenin mono (Figure 8D) and dihexoside (Figure 8A) conjugates, resulting from the flavonoid biosynthetic pathway, were low in the initial mature green fruits and remained largely unaffected in the dark treatment. The hexosides of the phenylpropanoids ferulic acid (Figure 8B), caffeic acid (Figure 8C) and sinapic acid (Figure 8F), and the flavonoid quercetin-3-glucoside (Figure 8E), were highest after the blue and white treatments, although the differences with other light treatments or darkness are not significant (*p* > 0.01) in all cases. In the overall comparison of the various light spectra, 29 LCMS compounds were found to be significantly higher and four lower (7.5% of total metabolites are responsive) in the blue compared to the far-red light treatment; five were higher and one lower in the red versus the far-red light treatment; 11 were higher and one lower (2.7% of total metabolites are responsive) in the blue compared to the red light treatment; and finally, two were higher and nine lower (2.5% of total metabolites are responsive) when comparing the green to the white light treatment (Table S7).

With respect to the 108 primary metabolites detected by untargeted GCMS, a PCA based on all samples indicated a clear effect of the seven days treatment time, and an effect of light versus dark exposure (Figure S2). These primary metabolites were similarly responsive in all comparisons: eight metabolites (7.4%) significantly different in the comparison of blue light treatment vs. far-red light treatment; three metabolites (2.8%) with blue light treatment vs. red light treatment; and five metabolites (4.6%) for green light treatment vs. white light treatment.

## 3. Discussion 

### 3.1. Light Affects Ripening-Related Processes

In previous papers, we showed that light intensity and spectral quality can affect the levels of ascorbic acid (vitamin C) and investigated the possible underlying physiological and biochemical mechanisms [16,18]. In the current work we expanded our research towards the effect of light on the global metabolome of tomato fruit pericarp, via means of comprehensive metabolomics technologies. We focused on metabolites related to visual quality (pigments), nutritional quality (vitamins and potential health-related compounds) and organoleptic characteristics (flavor-related compounds, sugars, acids).

The level of pigments, specifically lycopene, in the pericarp, is one of the key aspects in overall visual quality of red tomato fruits, as it refers to fruit ripeness. The increase of naringenin-chalcone during up to 12 days in light, followed by a decrease, is a typical ripening pattern for tomatoes [23]. This is a good indication that in light, green, mature, detached fruits follow their normal ripening-related changes in flavonoid biosynthesis, and therefore detached fruits may be used as model to study light effects on fruits during ripening on the vine. Exposing detached mature green tomatoes to white LED light for up to 15 days resulted in both an increase in all lipid-soluble carotenoids, including lycopene, and a faster decrease in chlorophyll as compared to exposing the fruits to darkness. This result suggests that ripening in terms of lycopene accumulation proceeds at a faster pace under light than in darkness. In contrast, fruit firmness progressed in a similar manner in both light and dark treatments [16]. Furthermore, significant differences between these light and dark treatments in fruit sucrose levels have not been observed [18]. These results indicate that not all aspects of fruit ripening are influenced by light. Fruits exposed to light do not simply ripen faster than fruits kept in darkness. Although there were statistically significant differences between various monochromatic LEDs in their efficiency to influence the levels of the various fruit pigments (Figure 6), the effects were only minor, with blue and far-red light being the most contrasting treatments. However, as in experiment 2 the overall applied light sum was lower than in experiment 1 (lower intensity and shorter duration), the effects of specific wavelengths may be more obvious when using higher light intensities or longer durations. Overall, it can be concluded that the presence of light accelerates specific ripening-related processes in tomato pigmentation.

Typically, with the progress of ripening and concomitantly to the color changes, a decrease in fruit firmness is usually observed, as a result of the hydrolysis of cell walls compounds [24]. In our experiments, chlorogenic acid, one of the side products in lignin biosynthesis [25], was similar in light and dark-exposed fruits. However, other phenolic compounds directly related to the lignin pathway such as sinapic acid were higher in the light-exposed fruits. As there were no macroscopic differences in fruit firmness between these light and darkness treatments [16], it is concluded that the light effects on the lignin pathway, if any, are insufficient to affect the firmness of the fruit.

### 3.2. The Nutritional Value of Tomato Fruits Is Upregulated by Light

The tomato is a good source for a variety of metabolites with assumed or potentially beneficial effects on human health. The fruits contain vitamin E (tocopherols) and carotenoids (provitamin A) such as lycopene, alpha and beta-carotene and the xanthophylls lutein and zeaxanthin. Apart from carotenoids being vitamin A precursors, a common function of the abovementioned metabolites is that they potentially act as antioxidants in the human body, which have been related to the prevention of certain diseases [26]. In plants, the major role of these molecules is to protect the plant cells from oxidative damage mediated by reactive oxygen species [27,28]. Reactive oxygen species (ROS) are mainly produced during photosynthesis and mitochondrial respiration. A higher light intensity usually results in a higher photosynthetic rate and thus potentially in a higher production rate of ROS. In the current research, treatment with light resulted in an increase of the abovementioned lipophilic antioxidants, as compared to keeping fruits in darkness (Figure 1). This response was generally observed up to day 9, at which time point the fruits entered the breaker stage and started breaking down chlorophyll; thus, fruit photosynthesis is expected to be reduced dramatically upon further fruit ripening [16]. This orchestrated increase in antioxidants upon exposure of mature green fruit to (LED) light is potentially a defense mechanism of the tissue against photosynthesis-related oxidative stress. 

Due to the specific absorbance spectrum of chlorophylls [29], the blue and red LED treatments may have resulted in higher photosynthetic rates with a likely concomitant effect on the ROS production in the green fruit as compared to both green and far-red LED. However, identifying the specific light-signaling effects appeared more complex. Addition of red or blue light (compared to far-red or green) each increased the contents of a limited number of lipophilic compounds with antioxidant functions in vivo (e.g., zeaxanthin and alpha-tocopherol), which was potentially due to an upregulation of the photosynthetic rate. Furthermore, antioxidants such as flavonoids (e.g., naringenin-hexoses), phenylpropanoids (e.g., caffeic acids and dicaffeoyl-quinic acids) and other phenolic compounds were also upregulated by blue light relative to other LED colors. These results suggest wavelength-specific light signaling effects. Therefore, specific photoreceptors are potentially involved in the observed differential effects of LED qualities on the compositions of antioxidants and other fruit metabolites.

Other health related metabolites typically found in tomato fruits were also increased in fruits exposed to light. Phenols such as naringenin, a flavanone type of flavonoid; rutin (or quercetin-3-O-rhamnosylglucoside), a flavonol type of flavonoid; caffeic acid, a dihydroxy cinnamate type of phenylpropanoid; and their derivatives, potentially have beneficial effects on human health presumably through their antioxidant function. Specifically, naringenin has been related to the prevention and treatment of Alzheimer’s disease and cancer [30], while phloretin, a dehydrochalcone type of flavonoid, exerts a pharmaceutical antioxidant function [31]. Light stimulated the accumulation of all abovementioned phenols in tomato pericarp, ranging from 4-fold for dicaffeoylquinic acid isomer III up to 350-fold for naringenin-C-diglycoside, as compared to fruits kept in darkness (Table S3). Another major antioxidant, ascorbic acid (vitamin C), was also higher (up to 4.8 times) in the light treatment [16]. Other metabolites for which various health effects have been suggested, such as L-threonate, increased in tomatoes when exposed to white light. Moreover, as glycoalkaloids significantly decreased upon exposure to light (Table S4), a potential negative effect of tomato consumption on the human intestine, i.e., aggravation of inflammatory bowel disease [32], is less likely in fruits treated with light compared to fruits kept in darkness. It is thus concluded that light can enhance the potential nutritional quality of tomato fruit.

### 3.3. Taste-Related Metabolites of Tomato Fruits Can Be Manipulated by Light

Studies employing both consumer and trained sensory panels, and metabolic measurements, define those taste aspects that are the main drivers for consumer preference. The appeal of tomato fruits is predominantly driven by the soluble carbohydrate content and to a lesser extent by the content of organic acids and texture [33]. However, to achieve a better prediction power in future modeling of taste and appeal, a higher level of complexity has to be integrated. For example, the various sugars found in tomatoes have a different impact on sweetness as sensed by the human tongue [34]. In the current research, no significant changes in taste-related sugars (sucrose) due to irradiance or light spectrum treatments have been observed. From the two most abundant organic acids in tomato and key to a sour taste, i.e., malic acid and citric acid [35], malic acid significantly decreased, by a factor 2.6, in the 15 day light treatment compared to darkness. As only the relative but not the absolute concentration of malic acid was quantified, the actual effect of its light-induced decrease is yet unknown but may potentially affect fruit taste. In parallel, a decreased (factor 2.4) of gamma-aminobutyric acid (GABA) was observed when fruits were treated with light. GABA is an inhibitory neurotransmitter that can also affect taste buds associated with the perception of sweet, bitter and umami [36]. Since GABA has been proposed to be an inhibitor of sweetness perception, light treatment of tomato fruit may have a positive effect on consumer preference by reducing the level of this sweetness inhibitor. Further research is essential in order to decipher the exact impact of this light-induced GABA alteration on tomato taste perception. In addition, glutamic acid increased substantially upon exposure to light, a result that potentially leads to the enhancement of the umami taste perception [37]. Naringenin also increased by light, which may have a potential effect on the perception of bitterness [38]. Guaiacol and methyl-salicylate (phenolic volatiles), present in tomato as non-volatile glycosides, can be released via de-glycosylation upon eating/cell disruption due to endogenous glycosidase activity, giving a characteristic smoky aroma [39]. Guaiacol-xylosyl-glucoside and methyl-salicylate-xylosyl-glucoside (xylosyl-glucose conjugates of guaiacol and methyl-salicylate, respectively) are well-known substrates for this glycosidase-induced release of smoky aroma [40]. Both compounds were detected in our fruits, but their relative levels were not different between light and dark-exposed fruits, nor between fruits exposed to different light qualities (data not shown). It is thus likely that the light environment did not influence the smoky aspect of the tomato aroma profile, at least in the variety used in these experiments.

## 4. Materials and Methods 

### 4.1. Plant Material

Tomato fruits (Solanum lycopersicum cv. Vimoso, average fruit weight 43 g) from a commercial glasshouse (Royal Pride Holland) in Middenmeer, the Netherlands (52°46′58″ N, 5°03′42″ E), were used. Trusses of 8 mature green fruits that were hanging on the same position on the plants were harvested and transported to Wageningen University and Research facilities in Wageningen, the Netherlands, within 3 h of harvest. The harvest took place between 8:00 and 10:00. To ensure a similar developmental stage, only fruits from positions 3 and 4 in the truss counting acropetally (from the oldest to the youngest fruits) were used. The selection of fruits was further narrowed down by measuring and selecting fruits of similar fresh weight (43 ± 3 g), size (6.2 ± 0.3 cm) and color (−0.6 ± 0.03 NAI and 0.07 ± 0.02 NDVI color indices; pigment analyzer spectroradiometer, PA1101, CP, Potsdam-Golm, Germany).

### 4.2. Light Treatments and Abiotic Environment

We performed two experiments to determine the effects of light intensity and light quality on the metabolite composition of tomato. In experiment 1 the overall effect of light was investigated by treating detached fruits with either white light or complete darkness for 15 days. In experiment 2, fruits were treated with different light spectra for seven days. The light treatments started in both experiments approximately 5 h after harvest. Both experiments have been previously described [16]. Experiment 1 took place in a different part of the year than experiment 2. Therefore, for both experiments, fruits were picked from a greenhouse compartment with artificial lighting so that the difference in growth irradiance was the minimum possible and the fruits of the two experiments had as similar as possible initial ascorbic acid levels. In both experiments, small compartments with an illuminated area of 80 cm × 50 cm were used for the application of the light treatments (2 compartments in experiment 1 and 6 compartments in experiment 2). Each compartment consisted of an aluminum frame that supported the LED module on top and was covered on the sides by highly reflective MC-PET sheets (SRF-A032T, Sekisui Plastics Co., LTD, Osaka, Japan) which significantly improved light distribution. This material was chosen for its ability to reflect light without affecting the spectrum, a property that was further verified by a spectrophotometer with the use of an integrated sphere in a dark box (USB-4000, Ocean Optics, Dunedin, FL, USA). The compartments were placed on the top of tables inside a climate cell, and each compartment represented an individual light treatment. The distance between the LED modules and the surface of the table was 80 cm. The fruits were placed in the middle of the compartment (in an area of 60 cm × 30 cm) where the light was most evenly distributed (less than 10 μmol m^−2^ s^−1^ difference between the brightest and darkest spots). In order to expose the fruits equally to the light, fruits were daily rotated within the illuminated area. Irradiance and spectral distribution were measured in all treatments on a 5 by 5 cm grid with a spectroradiometer (USB2000, Ocean Optics, Duiven, The Netherlands; calibrated against a standard light source). Light was continuously applied (24 h per day) in both experiments.

In experiment 1 the fruits were ripened for 15 days in complete darkness or under white light (broad spectrum) of 500 μmol m^−2^ s^−1^. White light was supplied with blue phosphorous-coated LEDs (GreenPower LED research module, Philips, Eindhoven, The Netherlands). In experiment 2 the fruits were treated with 4 different monochromatic spectra (blue, red, far-red and green) for seven days. The irradiance levels of the monochromatic spectra were set at 250 μmol m^−2^ s^−1^. In all monochromatic treatments there was a white background light of 100 μmol m^−2^ s^−1^ supplied by the same blue phosphorous-coated LEDs as used in experiment 1. Thus, the total irradiance in each treatment of experiment 2 was 350 μmol m^−2^ s^−1^. Red and blue monochromatic light had dominant wavelengths at 638 and 450 nm, respectively, and were supplied by Royal Blue and Red Luxeon K2 LEDs (Lumileds Lighting Company, San Jose, CA, USA). Far-red monochromatic light had a dominant wavelength at 740 nm and was supplied by GreenPower LED research module (Philips, Eindhoven, The Netherlands), while the green light was provided by custom made modules with a dominant wavelength at 520 nm (Philips prototype). Due to energy output limitations, the green light treatment consisted of only 150 μmol m^−2^ s^−1^ green light supplemented with 200 μmol m^−2^ s^−1^ of white light, yielding a total irradiance identical to the other treatments.

To ensure an even temperature distribution between and within the compartments, we used fans producing a turbulent air flow. The temperature in each compartment was logged with TC-08 data loggers (Picotechnology LTD., Cambridge, UK). During all treatments the relative air humidity was 70% and the CO_2_ concentration was between 352 and 428 ppm. The average temperature of the fruits was 18.5 °C (± 0.15 °C) in all treatments, as measured with k-type thermocouples (calibrated in freezing and boiling distilled water) attached on the epidermis at the lower side of the fruits to avoid direct shortwave radiation on the thermocouple. At the starts of treatments, the calyx of each fruit was removed and the fruits were placed with their calyx scars on the table plate, in order to minimize the rate of water loss. Water loss rates were similar between all treatments of the same experiment, as was established by frequently weighing the fruits (data not shown). In experiment 1 the fruits lost 2.4% of their initial weights, while in experiment 2 the fruits lost 0.62% of their initial weights. No significant differences in weight loss were observed between treatments within each experiment (data not shown). After the light treatments, 3 pericarp discs per fruit were removed from the equator of the fruits with a cork borer of 1 cm diameter. Only the outer pericarp and epidermis were included (no columella tissue). One sample, i.e., one biological replicate, consisted of a pool of 2 fruits (6 pericarp discs in total per pool). Upon dissection, the sample was flash frozen in liquid nitrogen. Each sample was then ground into a fine powder using liquid nitrogen. Samples were stored at −80 °C before metabolomics analyses.

### 4.3. Metabolomics Platforms—Extraction, Analysis and Data Processing

Metabolite extractions and analyses were all performed as recently described [41], using 100 mg fresh weight of frozen tomato pericarp powder per extraction. In short, lipid-soluble compounds including carotenoids, tocopherols and chlorophylls were extracted using water/chloroform/methanol with 0.1% butylhydroxytoluene as antioxidant and Sudan I as internal standard. The lipid phase was dried; compounds were re-dissolved in 500 µL ethylacetate and analyzed in a targeted manner by C30-reversed phase HPLC (Waters, Etten-Leur, The Netherlands) coupled to photodiode array (PDA) detection for both carotenoids and chlorophylls, and fluorescence (Fl) detection for tocopherols (in short: HPLC-PDA-Fl). These lipid-soluble compounds were quantified using calibration curves from authentic standards [42]. Semi-polar compounds, including flavonoids, phenylpropanoids, alkaloids and various volatile-glycosides, were extracted in 75% methanol containing 0.1% formic acid and by C18-reversed phase HPLC (Acquity system, Waters) coupled to both PDA detection (Waters 2996) and a LTQ-Orbitrap FTMS hybrid system (Thermo Scientific, Breda, The Netherlands) with electrospray ionization (ESI) in negative mode, a mass resolution of 70,000 FWHM and a m/z range of 90–1350 (in short: LCMS) [43]. Polar compounds, including sugars, organic acids and amino acids, were extracted using water–methanol–chloroform containing ribitol as the internal standard. The polar phase was dried; derivatized with both methoxyamine and N-methyl-N-(trimethylsilyl) trifluoroacetamide; and mixed with 1 µL of an alkane series (C10-C30) using an online derivatization/injection robot (CTC Analytics, Zwingen, Switzerland) and analyzed by an Agilent 6890 GC (Santa Clara, CA, USA) coupled to Pegasus III TOF MS (Leco Instruments, St. Joseph, MI, UDA) with 70 eV electron impact (EI) ionization (in short: GCMS) [44]. The unprocessed datasets from both the GCMS and LCMS platforms are available at https://doi.org/10.5061/dryad.5qfttdz2j. 

Samples from both experiment 1 and experiment 2 were all extracted and analyzed in a single series in a random order. The processing of data from the targeted HPLC-PDA-Fl analysis of lipid-soluble compounds (isoprenoids) was performed using Empower software (Waters); these lipid-soluble compounds were annotated and quantified using authentic standards. Both LC-MS and GC-MS data were processed in an untargeted manner, using Metalign software (version 3, Wageningen, Netherlands) [45] for automatic noise estimation, unbiased peak picking (with the intensity based on peak height) and alignment. Settings are provided in Figure S3. The resulting datasets were then further processed by grouping all mass features putatively derived from the same compound (mass clusters), based on their similarity in both retention time (scan number) and relative abundance across samples, thereby removing mass signal redundancy and retaining a single, representative value (i.e., the total of ion counts of clustered mass signals) per metabolite, using MSClust software (version 300817, Wageningen, The Netherlands) [46]. In cases when metabolite intensities did not exceed the detection threshold, their levels were assumed as zero. In addition, the resulting mass clusters actually represent low energy-ESI (pseudo) mass spectra in the case of LCMS and high energy-EI mass spectra in the case of GCMS; these mass spectra were used for annotation of selected compounds. For LCMS, the molecular ions of selected compounds were manually checked in the pseudo spectrum and annotated based on comparisons of retention time; accurate mass; isotopic pattern and (pseudo)mass spectrum information; and the corresponding PDA spectrum if available, with in-house databases [47] and on-line available metabolite databases such as KNApSAcK, HMDB and MassBank. For GC-MS, the mass spectra from MSClust and calculated retention indices, based on the retention of the added alkane series, was compared with available EI-spectral libraries such as the NIST2014 and the Golm spectral database (http://gmd.mpimp-golm.mpg.de/), and an in-house library of derivatized standards. 

### 4.4. Statistical Analysis

Unsupervised multivariate analysis (principal component analysis, or PCA; GeneMaths XT, Applied Maths, Sint-Martens-Latem, Belgium) of LCMS and GCMS datasets, the latter after correction for variation in the internal standard ribitol, was performed to visualize global differences between samples. Metabolite intensities resulting from the untargeted LCMS and GCMS analyses (Tables S1 and S2) were firstly log2-transformed and then normalized to univariance. Whether treatments differed significantly was tested with the calculation of the Least Significant Difference (LSD). Analysis of variance (ANOVA) was used to test either the effect of the light treatment or to compare the initial and final levels of metabolites across treatments. The pool of six pericarp discs from two fruits (three discs per fruit) was considered an independent replicate. Three replicates (three pools of two fruits each) per treatment were measured. Considering each sample as an independent replicate may have underestimated the random variance. Therefore, the multiple comparisons (Tukey’s test) have been conducted at α = 0.01 instead of the more commonly used α = 0.05.

## 5. Conclusions

Irradiance of detached fruit with white LED light significantly influenced the metabolic profiles of tomato pericarps, and influencing the light spectrum by additional illumination using colored LEDs had a more narrow range of effects. Exposure to light accelerated ripening in terms of accumulation of both pigments and flavonoids, and a reduction of alkaloids in the pericarp. Light positively influenced the levels of a variety of health related compounds, such as tocopherols, carotenoids and phenolic compounds, next to the previously reported increase in vitamin C, thereby potentially enhancing the nutritional value of these fruits. Light also significantly influenced the levels of some well-known flavor-related compounds, such as malic acid, glutamate and GABA, but not the levels of smoky-aroma precursors. The effects of specific LED light spectra on the metabolome exerted diverse effects with additional blue light being the most effective on both pigments and other secondary metabolites. The results of this study provide, for the first time, a more detailed insight in the effects of LED light on tomato fruits and how light may affect the quality of tomato fruits.

## Supplementary Materials

The following are available online at https://www.mdpi.com/2218-1989/10/6/266/s1. Table S1: Total LCMS metabolites. Table S2: Total GCMS metabolites. Table S3: LCMS metabolites experiment 1 light and dark. Table S4: LCMS metabolites experiment 1 time. Table S5: GCMS metabolites experiment 2 light and dark. Table S6: GCMS metabolites experiment 2 time. Table S7: LCMS metabolites experiment 2 time. Figure S1: LCMS PCA experiment 2. Figure S2: GCMS PCA experiment 2. Figure S3: Metalign settings.
