# Supplementary material for: Modulation of the Tomato Fruit Metabolome by LED Light
Source: Metabolites. 2020 Jun 26;10(6):266. doi: 10.3390/metabo10060266 (PMC7345426; doi:10.3390/metabo10060266)

Supplemental Material

Supplemental Table 3: Responsive annotated metabolites detected in tomato pericarp by liquid chromatography mass spectroscopy (LC-MS), when fruits were treated with light (500 μmol m^-2^ s^-1^) vs darkness for 15 days (Exp.1; α=0.01, n=3).

|  |  |  |  |  |  |
| --- | --- | --- | --- | --- | --- |
| **Retention Time (min)** | **Mass** | **Metabolite Name** | **Response to treatment (times increase between light and dark at 15 days)** | **Level of annotation** | **T-test** |
| 13.42 | 595.17 | Naringenin-C-diglycoside | 349.63 | 2 | 0.001 |
| 30.52 | 479.12 | unknown | 165.80 |  | 0.008 |
| 31.96 | 459.09 | unknown | 100.29 |  | <0.001 |
| 15.63 | 223.06 | Sinapic acid | 77.91 | 2 | <0.001 |
| 21.36 | 203.06 | unknown | 70.37 |  | <0.001 |
| 25.74 | 587.20 | unknown | 65.61 |  | <0.001 |
| 32.48 | 819.27 | unknown | 58.70 |  | <0.001 |
| 31.62 | 445.08 | unknown | 58.68 |  | 0.001 |
| 32.70 | 425.14 | unknown | 57.87 |  | <0.001 |
| 19.24 | 433.11 | unknown | 50.66 |  | 0.009 |
| 13.78 | 351.13 | unknown | 48.94 |  | 0.001 |
| 20.89 | 577.25 | N307 | 44.88 | 4 | <0.001 |
| 25.47 | 415.20 | unknown | 43.52 |  | 0.009 |
| 33.58 | 287.06 | unknown | 41.71 |  | <0.001 |
| 21.34 | 333.06 | unknown | 41.12 |  | <0.001 |
| 22.60 | 577.25 | C26H42O14 | 39.30 |  | 0.002 |
| 24.95 | 283.06 | unknown | 38.48 |  | 0.004 |
| 25.92 | 449.11 | unknown | 36.38 |  | 0.004 |
| 22.91 | 597.18 | Phloretin-C-diglycoside | 34.68 | 2 | 0.003 |
| 13.04 | 309.12 | unknown | 29.58 |  | <0.001 |
| 28.96 | 855.25 | unknown | 28.45 |  | 0.002 |
| 29.71 | 429.14 | unknown | 27.62 |  | 0.002 |
| 29.57 | 283.06 | unknown | 26.45 |  | <0.001 |
| 22.37 | 245.09 | unknown | 25.42 |  | <0.001 |
| 44.11 | 855.31 | unknown | 24.80 |  | <0.001 |
| 38.86 | 542.15 | unknown | 23.67 |  | <0.001 |
| 23.46 | 335.17 | unknown | 23.23 |  | <0.001 |
| 17.28 | 759.23 | unknown | 22.22 |  | 0.009 |
| 29.28 | 511.18 | unknown | 19.66 |  | 0.003 |
| 26.82 | 433.11 | Naringenin-hexose | 19.24 |  | 0.003 |
| 35.70 | 623.18 | unknown | 18.49 |  | 0.001 |
| 35.75 | 775.28 | unknown | 17.71 |  | 0.002 |
| 34.19 | 623.18 | unknown | 17.17 |  | <0.001 |
| 39.90 | 271.06 | naringenin | 15.83 | 1 | <0.001 |
| 25.58 | 283.06 | unknown | 15.72 |  | 0.004 |
| 35.79 | 923.28 | unknown | 15.14 |  | <0.001 |
| 39.94 | 431.90 | unknown | 14.99 |  | <0.001 |
| 27.02 | 1120.52 | unknown | 14.03 |  | 0.006 |
| 30.58 | 221.07 | unknown | 13.94 |  | 0.003 |
| 20.25 | 367.10 | 3-O-Feruloylquinic acid | 12.22 | 2 | <0.001 |
| 23.39 | 577.25 | C26H42O14 | 12.01 | 4 | <0.001 |
| 7.81 | 371.06 | unknown | 11.87 |  | 0.003 |
| 2.30 | 351.06 | unknown | 11.83 |  | 0.009 |
| 12.59 | 351.07 | unknown | 11.81 |  | <0.001 |
| 36.94 | 677.15 | 3,4,5-Tricaffeoylquinic acid | 11.19 | 1 | 0.002 |
| 24.29 | 433.11 | Naringenin-hexose | 10.71 | 2 | 0.004 |
| 31.89 | 221.07 | unknown | 10.19 |  | 0.004 |
| 32.03 | 677.28 | N713 | 10.10 | 4 | 0.007 |
| 24.83 | 415.20 | unknown | 9.38 |  | 0.001 |
| 14.57 | 351.07 | unknown | 8.53 |  | 0.001 |
| 28.10 | 515.12 | dicaffeoylquinic acid IV | 7.49 |  | 0.003 |
| 40.39 | 271.06 | naringenin-chalcone | 7.43 | 1 | 0.009 |
| 22.06 | 405.18 | unknown | 7.41 |  | 0.006 |
| 39.04 | 677.15 | unknown | 6.92 |  | <0.001 |
| 26.39 | 415.20 | unknown | 6.84 |  | 0.002 |
| 25.62 | 515.12 | dicaffeoylquinic acid II | 6.75 | 2 | 0.004 |
| 21.04 | 662.18 | unknown | 6.42 |  | 0.008 |
| 2.16 | 175.02 | Ascorbic acid | 6.15 | 2 | 0.003 |
| 4.81 | 345.08 | unknown | 5.85 |  | 0.007 |
| 38.30 | 677.15 | Tricaffeoylquinic acid II | 5.72 | 2 | <0.001 |
| 1.96 | 326.11 | unknown | 5.17 |  | <0.001 |
| 2.03 | 535.15 | unknown | 5.11 |  | 0.003 |
| 20.32 | 391.16 | unknown | 4.58 |  | 0.008 |
| 12.66 | 353.09 | caffeoylquinic acid | 4.44 | 3 | 0.004 |
| 21.77 | 525.52 | unknown | 4.44 |  | 0.002 |
| 32.25 | 515.12 | dicaffeoylquinic acid V | 4.34 | 2 | <0.001 |
| 21.70 | 609.15 | quercetin-3-O-rutinoside (rutin) | 4.34 | 1 | 0.007 |
| 21.65 | 345.15 | unknown | 4.27 |  | <0.001 |
| 17.44 | 1272.58 | unknown | 4.24 |  | 0.006 |
| 26.68 | 515.12 | dicaffeoylquinic acid III | 3.99 |  | 0.001 |
| 32.30 | 411.20 | unknown | 3.44 |  | 0.010 |
| 27.18 | 415.20 | unknown | 3.10 |  | 0.004 |
| 2.09 | 135.03 | L-Threonate | 2.75 | 3 | 0.003 |
| 3.73 | 286.97 | unknown | 2.59 |  | 0.003 |
| 34.82 | 1180.54 | unknown | 2.42 |  | 0.004 |
| 32.55 | 845.21 | unknown | 2.40 |  | 0.007 |
| 1.86 | 331.05 | unknown | 2.15 |  | 0.007 |
| 2.61 | 333.06 | unknown | 2.07 |  | 0.007 |
| 2.01 | 195.05 | unknown | 1.53 |  | 0.008 |
| 2.63 | 317.05 | unknown | 0.53 |  | 0.001 |
| 40.57 | 539.25 | unknown | 0.45 |  | 0.003 |
| 1.90 | 214.05 | unknown | 0.31 |  | 0.001 |
| 30.99 | 917.23 | unknown | 0.13 |  | <0.001 |
|  |  |  |  |  |  |

Supplemental Table 4: Responsive annotated metabolites detected in tomato pericarp by liquid chromatography mass spectroscopy (LC-MS), when fruits were treated with light (500 μmol m^-2^ s^-1^) for 15 days compared to initial levels (Exp.1; α=0.01, n=3).

|  |  |  |  |  |  |
| --- | --- | --- | --- | --- | --- |
| **Retention Time (min)** | **Mass** | **Metabolite Name** | **Response to treatment (times increase between 15 and 0 days in light)** | **Level of annotation** | **t-test** |
| 28.10 | 515.12 | dicaffeoylquinic acid IV | 1518.54 |  | 0.002 |
| 32.03 | 677.28 | N713 | 1130.41 | 4 | 0.003 |
| 39.90 | 271.06 | naringenin | 619.00 | 1 | <0.001 |
| 36.94 | 677.15 | 3,4,5-Tricaffeoylquinic acid | 596.26 | 1 | 0.001 |
| 22.37 | 245.09 | unknown | 557.21 |  | <0.001 |
| 12.66 | 353.09 | caffeoylquinic acid | 478.72 | 3 | <0.001 |
| 20.89 | 577.25 | N307 | 454.79 | 4 | <0.001 |
| 22.60 | 577.25 | C26H42O14 | 380.62 |  | 0.002 |
| 13.42 | 595.17 | Naringenin-C-diglycoside | 364.57 | 2 | 0.001 |
| 25.62 | 515.12 | dicaffeoylquinic acid II | 351.29 | 2 | 0.002 |
| 26.75 | 839.33 | unknown | 331.50 |  | 0.002 |
| 40.39 | 271.06 | naringenin-chalcone | 331.43 | 1 | 0.003 |
| 32.25 | 515.12 | dicaffeoylquinic acid V | 165.62 | 2 | <0.001 |
| 31.96 | 459.09 | unknown | 158.12 |  | <0.001 |
| 24.29 | 433.11 | Naringenin-hexose | 157.07 | 2 | 0.002 |
| 20.19 | 667.80 | unknown | 155.39 |  | <0.001 |
| 15.23 | 491.18 | N231 | 150.30 | 4 | 0.002 |
| 23.39 | 577.25 | C26H42O14 | 144.52 | 4 | <0.001 |
| 22.98 | 760.82 | double charged: >1500 D | 137.72 | 4 | <0.001 |
| 27.02 | 1120.52 | unknown | 131.74 |  | 0.004 |
| 24.95 | 283.06 | unknown | 127.38 |  | 0.003 |
| 38.30 | 677.15 | Tricaffeoylquinic acid II | 127.21 | 2 | <0.001 |
| 23.77 | 328.65 | double charged: >1500 D | 126.49 | 4 | <0.001 |
| 22.19 | 760.82 | unknown | 120.10 |  | <0.001 |
| 26.82 | 433.11 | Naringenin-hexose | 115.21 |  | 0.002 |
| 24.23 | 291.14 | unknown | 109.14 |  | 0.002 |
| 13.04 | 309.12 | unknown | 103.31 |  | <0.001 |
| 40.45 | 334.06 | unknown | 99.49 |  | <0.001 |
| 21.00 | 328.65 | unknown | 95.82 |  | <0.001 |
| 29.05 | 855.33 | unknown | 95.80 |  | 0.003 |
| 25.92 | 449.11 | unknown | 93.35 |  | 0.004 |
| 26.68 | 515.12 | dicaffeoylquinic acid III | 89.05 |  | <0.001 |
| 30.52 | 479.12 | unknown | 87.67 |  | 0.008 |
| 32.03 | 694.28 | unknown | 84.63 |  | <0.001 |
| 39.94 | 431.90 | unknown | 84.57 |  | <0.001 |
| 15.63 | 223.06 | Sinapic acid | 83.29 | 2 | <0.001 |
| 39.04 | 677.15 | unknown | 82.61 |  | <0.001 |
| 9.90 | 341.09 | caffeic acid hexose III | 81.76 | 2 | <0.001 |
| 18.72 | 189.08 | unknown | 80.98 |  | 0.004 |
| 16.13 | 295.14 | unknown | 74.05 |  | <0.001 |
| 33.58 | 287.06 | unknown | 72.58 |  | <0.001 |
| 11.42 | 175.06 | unknown | 72.17 |  | <0.001 |
| 39.20 | 821.32 | unknown | 72.05 |  | 0.002 |
| 34.08 | 691.26 | unknown | 70.51 |  | 0.006 |
| 25.74 | 587.20 | unknown | 69.66 |  | <0.001 |
| 21.36 | 203.06 | unknown | 66.73 |  | <0.001 |
| 30.70 | 693.28 | unknown | 65.18 |  | 0.006 |
| 26.59 | 505.13 | unknown | 62.44 |  | <0.001 |
| 31.62 | 445.08 | unknown | 60.58 |  | 0.001 |
| 13.78 | 351.13 | unknown | 60.49 |  | 0.001 |
| 32.48 | 819.27 | unknown | 59.49 |  | <0.001 |
| 2.08 | 193.04 | galacturonate/glucuronate | 57.74 | 3 | <0.001 |
| 32.70 | 425.14 | unknown | 57.51 |  | <0.001 |
| 40.30 | 431.90 | unknown | 57.28 |  | <0.001 |
| 17.28 | 759.23 | unknown | 56.71 |  | 0.008 |
| 24.97 | 503.12 | unknown | 52.37 |  | <0.001 |
| 25.80 | 581.15 | unknown | 51.63 |  | 0.001 |
| 2.30 | 351.06 | unknown | 50.42 |  | 0.006 |
| 13.17 | 593.24 | unknown | 49.71 |  | <0.001 |
| 12.59 | 351.07 | unknown | 49.19 |  | <0.001 |
| 34.82 | 1180.54 | unknown | 48.05 |  | <0.001 |
| 30.58 | 221.07 | unknown | 47.87 |  | 0.002 |
| 17.44 | 1272.58 | unknown | 47.28 |  | <0.001 |
| 20.32 | 391.16 | unknown | 47.04 |  | <0.001 |
| 19.24 | 433.11 | unknown | 45.71 |  | 0.009 |
| 39.51 | 867.33 | unknown | 42.96 |  | 0.001 |
| 25.47 | 415.20 | unknown | 42.60 |  | 0.009 |
| 22.91 | 597.18 | Phloretin-C-diglycoside | 40.25 | 2 | 0.003 |
| 10.24 | 327.11 | unknown | 40.23 |  | 0.002 |
| 12.75 | 817.20 | unknown | 40.00 |  | 0.006 |
| 21.34 | 333.06 | unknown | 39.62 |  | <0.001 |
| 21.04 | 662.18 | unknown | 38.48 |  | 0.001 |
| 11.35 | 353.09 | 5-Caffeoyl-quinic acid | 36.32 | 2 | <0.001 |
| 23.46 | 335.17 | unknown | 34.11 |  | <0.001 |
| 29.28 | 511.18 | unknown | 33.99 |  | 0.003 |
| 17.07 | 295.05 | unknown | 33.90 |  | <0.001 |
| 34.60 | 1150.53 | unknown | 33.68 |  | 0.005 |
| 9.03 | 341.09 | caffeic acid hexose II | 32.75 | 2 | <0.001 |
| 28.96 | 855.25 | unknown | 30.19 |  | 0.002 |
| 29.57 | 283.06 | unknown | 29.45 |  | <0.001 |
| 5.24 | 378.13 | unknown | 28.97 |  | <0.001 |
| 29.71 | 429.14 | unknown | 28.77 |  | 0.002 |
| 19.94 | 433.11 | Naringenin-hexose | 28.19 |  | 0.009 |
| 4.81 | 345.08 | unknown | 27.95 |  | 0.003 |
| 23.05 | 471.19 | unknown | 26.87 |  | 0.002 |
| 44.11 | 855.31 | unknown | 25.92 |  | <0.001 |
| 2.03 | 535.15 | unknown | 23.95 |  | <0.001 |
| 11.05 | 341.09 | caffeic acid hexose IV | 23.69 | 2 | <0.001 |
| 38.86 | 542.15 | unknown | 23.09 |  | <0.001 |
| 40.14 | 805.33 | unknown | 22.27 |  | 0.009 |
| 43.02 | 1009.45 | unknown | 21.94 |  | <0.001 |
| 35.70 | 623.18 | unknown | 19.51 |  | 0.001 |
| 35.75 | 775.28 | unknown | 18.98 |  | 0.002 |
| 11.12 | 343.10 | unknown | 18.49 |  | 0.002 |
| 24.83 | 415.20 | unknown | 17.87 |  | <0.001 |
| 24.18 | 371.11 | unknown | 17.29 |  | 0.002 |
| 23.21 | 336.15 | unknown | 17.22 |  | 0.002 |
| 2.16 | 175.02 | Ascorbic acid | 16.84 | 2 | <0.001 |
| 15.45 | 797.31 | N178 | 16.33 | 4 | 0.002 |
| 19.37 | 1272.58 | Esculeoside B + FA | 16.32 | 2 | <0.001 |
| 39.56 | 857.30 | unknown | 16.31 |  | 0.002 |
| 25.17 | 903.22 | unknown | 16.22 |  | <0.001 |
| 35.79 | 923.28 | unknown | 16.19 |  | <0.001 |
| 17.03 | 569.21 | unknown | 16.13 |  | 0.004 |
| 31.89 | 221.07 | unknown | 16.09 |  | 0.004 |
| 34.19 | 623.18 | unknown | 15.43 |  | <0.001 |
| 25.58 | 283.06 | unknown | 15.19 |  | 0.004 |
| 30.65 | 871.23 | unknown | 14.92 |  | 0.003 |
| 24.52 | 1017.38 | unknown | 14.46 |  | 0.008 |
| 20.07 | 748.82 | unknown | 14.26 |  | <0.001 |
| 25.04 | 686.79 | unknown | 13.45 |  | 0.008 |
| 1.96 | 326.11 | unknown | 12.44 |  | <0.001 |
| 30.49 | 688.29 | unknown | 12.43 |  | <0.001 |
| 7.81 | 371.06 | unknown | 12.27 |  | 0.003 |
| 20.66 | 760.82 | unknown | 11.94 |  | 0.002 |
| 28.51 | 887.22 | unknown | 11.40 |  | <0.001 |
| 22.06 | 405.18 | unknown | 11.26 |  | 0.005 |
| 28.71 | 1152.54 | unknown | 10.64 |  | 0.008 |
| 35.41 | 688.29 | unknown | 9.95 |  | 0.001 |
| 14.57 | 351.07 | unknown | 9.95 |  | <0.001 |
| 25.29 | 1150.53 | unknown | 9.58 |  | 0.004 |
| 8.60 | 371.10 | unknown | 9.30 |  | 0.004 |
| 26.39 | 415.20 | unknown | 8.44 |  | 0.002 |
| 15.02 | 355.10 | Ferulic acid-hexose II | 7.82 | 2 | 0.004 |
| 3.52 | 284.01 | unknown | 7.49 |  | <0.001 |
| 16.04 | 427.18 | unknown | 7.42 |  | 0.008 |
| 21.77 | 525.52 | unknown | 7.32 |  | 0.002 |
| 21.65 | 345.15 | unknown | 6.90 |  | <0.001 |
| 1.86 | 331.05 | unknown | 6.47 |  | <0.001 |
| 14.53 | 385.11 | Sinapic acid-hexose | 5.93 |  | 0.003 |
| 32.30 | 411.20 | unknown | 5.13 |  | 0.006 |
| 20.25 | 367.10 | 3-O-Feruloylquinic acid | 5.13 | 2 | <0.001 |
| 21.97 | 471.19 | unknown | 5.07 |  | 0.007 |
| 14.01 | 355.10 | Ferulic acid-hexose I | 5.06 | 2 | <0.001 |
| 14.39 | 335.13 | unknown | 4.96 |  | <0.001 |
| 15.92 | 567.19 | dimethyl-dihydroxyphenyl hexoside | 4.74 | 3 | 0.002 |
| 24.65 | 1110.53 | unknown | 4.71 |  | 0.002 |
| 29.80 | 965.29 | unknown | 3.89 |  | 0.005 |
| 32.55 | 845.21 | unknown | 3.82 |  | 0.003 |
| 32.34 | 921.26 | unknown | 3.65 |  | 0.006 |
| 2.95 | 515.06 | unknown | 3.42 |  | 0.005 |
| 13.96 | 329.09 | 4-Hydroxy-2-(hydroxymethyl)benzoic acid-hexoside | 3.37 | 3 | <0.001 |
| 14.12 | 341.09 | caffeic acid-hexose V | 3.27 | 2 | <0.001 |
| 21.70 | 609.15 | quercetin-3-O-rutinoside (rutin) | 3.21 | 1 | 0.007 |
| 19.08 | 403.16 | unknown | 3.08 |  | 0.002 |
| 1.92 | 146.05 | L-Glutamic acid | 3.06 | 1 | <0.001 |
| 2.61 | 333.06 | unknown | 3.05 |  | <0.001 |
| 27.18 | 415.20 | unknown | 2.99 |  | 0.003 |
| 21.22 | 359.13 | C10H14O4-hexoside | 2.96 | 3 | 0.002 |
| 3.73 | 286.97 | unknown | 2.59 |  | 0.003 |
| 2.54 | 221.03 | unknown | 2.37 |  | <0.001 |
| 2.16 | 259.02 | unknown | 2.31 |  | 0.002 |
| 20.41 | 471.19 | N317 | 2.20 | 4 | 0.005 |
| 15.79 | 431.19 | N238 | 2.17 | 4 | 0.003 |
| 2.01 | 195.05 | unknown | 1.40 |  | 0.005 |
| 2.21 | 451.05 | unknown | 1.38 |  | 0.005 |
| 3.77 | 565.05 | UDP-glucose | 0.73 | 1 | 0.002 |
| 23.66 | 1094.54 | Hydroxytomatine III + FA | 0.66 | 2 | 0.008 |
| 22.33 | 1094.54 | Hydroxytomatine II + FA | 0.56 | 2 | <0.001 |
| 2.41 | 455.10 | unknown | 0.51 |  | 0.006 |
| 3.82 | 606.07 | UDP-N-acetyl-hexose amine II | 0.46 | 3 | 0.003 |
| 2.28 | 133.01 | malic acid | 0.45 | 1 | 0.002 |
| 1.82 | 145.06 | L-Glutamine | 0.43 | 1 | 0.003 |
| 2.65 | 184.99 | unknown | 0.42 |  | 0.001 |
| 2.63 | 317.05 | unknown | 0.41 |  | <0.001 |
| 2.01 | 341.11 | sucrose | 0.31 | 1 | 0.008 |
| 31.33 | 1136.55 | Acetoxy-tomatine III + FA | 0.23 | 2 | 0.001 |
| 26.86 | 1096.55 | N514_C50H85NO22 + FA | 0.22 |  | 0.001 |
| 1.95 | 325.12 | unknown | 0.17 |  | <0.001 |
| 29.64 | 1134.53 | unknown | 0.17 |  | 0.004 |
| 30.47 | 1078.54 | alpha-Tomatin + FA | 0.12 | 1 | <0.001 |
| 29.32 | 1076.53 | dehydrotomatin + FA | 0.12 | 2 | 0.001 |
| 2.03 | 549.17 | unknown | 0.11 |  | 0.005 |
| 1.90 | 214.05 | unknown | 0.11 |  | <0.001 |
| 30.63 | 1136.55 | Acetoxy-tomatine II + FA | 0.10 |  | <0.001 |
| 29.84 | 1108.55 | unknown | 0.08 |  | <0.001 |
| 29.98 | 1078.54 | alpha-Tomatin isomer + FA | 0.08 | 2 | 0.001 |
| 30.43 | 1069.52 | unknown | 0.08 |  | <0.001 |
| 30.34 | 1128.52 | unknown | 0.07 |  | <0.001 |
| 31.10 | 1076.53 | unknown | 0.05 |  | <0.001 |
| 29.91 | 1109.56 | unknown | 0.05 |  | <0.001 |
| 30.38 | 1136.55 | Acetoxy-tomatine I + FA | 0.05 |  | <0.001 |
| 21.16 | 1094.54 | Hydroxytomatine I + FA | 0.05 | 2 | <0.001 |
| 33.94 | 1150.56 | unknown | 0.04 |  | 0.001 |
| 31.55 | 1048.53 | unknown | 0.03 |  | 0.002 |
| 43.50 | 407.26 | unknown | 0.02 |  | <0.001 |
| 2.47 | 269.05 | unknown | 0.01 |  | <0.001 |
|  |  |  |  |  |  |

Supplemental Table 5: Responsive metabolites detected in tomato pericarp by GC-MS, after fruits were treated with light (500 μmol m^-2^ s^-1^) compared to treatment with darkness for 15 days (Exp.2; α=0.01, n=3).

|  |  |  |  |  |
| --- | --- | --- | --- | --- |
| **Retention Time (min)** | **Mass** | **Metabolite Name** | **Response to treatment (times increase between light and darkness at 15 days)** | **T-test** |
| 19.24 | 192 | Unknown | 528.56 | <0.001 |
| 19.18 | 163 | Unknown | 309.17 | <0.001 |
| 19.20 | 82 | Unknown | 224.53 | <0.001 |
| 19.45 | 73 | Unknown | 224.00 | <0.001 |
| 20.34 | 103 | Unknown | 155.50 | 0.003 |
| 21.97 | 189 | Unknown | 74.87 | <0.001 |
| 16.52 | 131 | Unknown | 30.93 | <0.001 |
| 21.96 | 333 | Unknown | 24.71 | 0.002 |
| 13.20 | 74 | Unknown | 13.78 | 0.008 |
| 16.50 | 173 | Unknown | 8.53 | <0.001 |
| 10.28 | 61 | Unknown | 8.39 | <0.001 |
| 17.60 | 333 | Unknown | 6.90 | 0.002 |
| 17.39 | 115 | Galacturonic acid | 4.69 | 0.007 |
| 6.20 | 166 | Unknown | 3.94 | <0.001 |
| 13.98 | 363 | Glutamic acid | 3.73 | 0.001 |
| 14.32 | 73 | Xylose | 2.54 | <0.001 |
| 17.90 | 147 | Unknown | 1.99 | 0.012 |
| 14.40 | 73 | Unknown | 1.77 | 0.010 |
| 18.83 | 221 | myo-inositol | 1.63 | 0.005 |
| 12.92 | 129 | GABA | 0.43 | 0.004 |
| 12.37 | 350 | Malic acid | 0.38 | <0.001 |
|  |  |  |  |  |

Supplemental Table 6: Responsive metabolites detected in tomato pericarp by GC-MS, after fruits were treated with light (500 μmol m^-2^ s^-1^) for 15 days compared to initial levels (Exp.2; α=0.01, n=3).

|  |  |  |  |  |
| --- | --- | --- | --- | --- |
| **Retention Time (min)** | **Mass** | **Metabolite Name** | **Response to treatment (times increase between 15 and 0 days in light)** | **T-test** |
| 17.60 | 333 | Unknown | 716.23 | <0.001 |
| 21.96 | 333 | Unknown | 421.25 | 0.002 |
| 21.97 | 189 | Unknown | 80.21 | <0.001 |
| 10.28 | 61 | Unknown | 37.25 | <0.001 |
| 17.39 | 115 | Galacturonic acid | 36.80 | <0.001 |
| 16.52 | 131 | Unknown | 28.08 | <0.001 |
| 13.98 | 363 | Glutamic acid | 17.45 | <0.001 |
| 12.98 | 84 | Glutamic acid | 9.62 | <0.001 |
| 14.32 | 73 | Xylose | 5.98 | <0.001 |
| 16.50 | 173 | Unknown | 5.71 | <0.001 |
| 6.20 | 166 | Unknown | 5.48 | <0.001 |
| 14.40 | 73 | Unknown | 4.18 | 0.001 |
| 15.32 | 86 | Unknown | 2.65 | 0.003 |
| 12.77 | 148 | Asparagine | 2.57 | 0.006 |
| 17.90 | 147 | Unknown | 1.95 | 0.009 |
| 15.52 | 147 | Unknown | 1.49 | 0.006 |
| 7.66 | 59 | Unknown | 0.68 | <0.001 |
| 12.92 | 129 | GABA | 0.36 | 0.005 |
| 8.72 | 144 | Unknown | 0.26 | 0.007 |
| 12.37 | 350 | Malic acid | 0.23 | <0.001 |
| 11.63 | 86 | Unknown | 0.22 | 0.004 |
| 23.77 | 133 | Unknown | 0.10 | <0.001 |
|  |  |  |  |  |

Supplemental Table 7: Effect of light spectrum during treatment of tomato fruits on metabolites detected by LC-MS. Fruits were treated for 7 days with a combination of monochromatic light (250 μmol m^-2^ s^-1^ for blue, red and far-red and 200 μmol m^-2^ s^-1^ for green treatments) and background broadband light (100 μmol m^-2^ s^-1^ for blue, red and far red and 150 μmol m^-2^ s^-1^ for green treatments; Exp. 2; α=0.01, n=3).

|  |  |  |  |  |  |  |
| --- | --- | --- | --- | --- | --- | --- |
| **Treatments Compared** | **Retention Time (min)** | **Mass** | **Metabolite Name** | **Response to treatment (times increase between the first and second light treatment at 15 days)** | **Level of annotation** | **T-test** |
| Blue VS Far Red | 13.49 | 179.03 | unknown | 29.01 |  | 0.002 |
| Blue VS Far Red | 21.34 | 333.06 | unknown | 15.68 |  | <0.001 |
| Blue VS Far Red | 31.78 | 435.13 | unknown | 10.82 |  | 0.003 |
| Blue VS Far Red | 28.98 | 481.13 | unknown | 9.18 |  | 0.003 |
| Blue VS Far Red | 26.46 | 451.12 | unknown | 7.88 |  | 0.007 |
| Blue VS Far Red | 12.63 | 171.03 | unknown | 7.72 |  | 0.003 |
| Blue VS Far Red | 15.34 | 351.07 | unknown | 7.53 |  | 0.002 |
| Blue VS Far Red | 14.57 | 351.07 | unknown | 7.45 |  | <0.001 |
| Blue VS Far Red | 16.53 | 351.07 | unknown | 7.42 |  | 0.002 |
| Blue VS Far Red | 12.59 | 351.07 | unknown | 6.97 |  | 0.001 |
| Blue VS Far Red | 26.37 | 433.11 | Naringenin-hexose | 6.66 | 2 | 0.003 |
| Blue VS Far Red | 27.13 | 495.11 | unknown | 5.32 |  | 0.010 |
| Blue VS Far Red | 19.24 | 433.11 | unknown | 5.00 |  | 0.001 |
| Blue VS Far Red | 19.94 | 433.11 | Naringenin-hexose | 4.86 |  | 0.003 |
| Blue VS Far Red | 4.81 | 345.08 | unknown | 3.34 |  | <0.001 |
| Blue VS Far Red | 22.04 | 449.11 | unknown | 2.92 |  | 0.003 |
| Blue VS Far Red | 14.53 | 385.11 | Sinapic acid-hexose | 2.50 |  | 0.002 |
| Blue VS Far Red | 25.92 | 449.11 | unknown | 2.48 |  | 0.002 |
| Blue VS Far Red | 22.60 | 577.25 | C26H42O14 | 2.45 |  | 0.005 |
| Blue VS Far Red | 28.96 | 855.25 | unknown | 2.44 |  | 0.001 |
| Blue VS Far Red | 13.42 | 595.17 | Naringenin-C-diglycoside | 2.20 | 2 | <0.001 |
| Blue VS Far Red | 9.03 | 341.09 | caffeic acid hexose II | 1.79 | 2 | 0.002 |
| Blue VS Far Red | 14.01 | 355.10 | Ferulic acid-hexose I | 1.78 | 2 | 0.005 |
| Blue VS Far Red | 31.62 | 445.08 | unknown | 1.72 |  | 0.008 |
| Blue VS Far Red | 15.02 | 355.10 | Ferulic acid-hexose II | 1.58 | 2 | 0.005 |
| Blue VS Far Red | 1.96 | 326.11 | unknown | 1.52 |  | 0.004 |
| Blue VS Far Red | 24.29 | 433.11 | Naringenin-hexose | 1.47 | 2 | 0.008 |
| Blue VS Far Red | 28.10 | 515.12 | dicaffeoylquinic acid IV | 1.38 |  | 0.009 |
| Blue VS Far Red | 3.77 | 565.05 | UDP-glucose | 1.33 | 1 | 0.004 |
| Blue VS Far Red | 22.98 | 760.82 | double charged: >1500 D | 0.74 | 4 | 0.002 |
| Blue VS Far Red | 1.90 | 214.05 | unknown | 0.59 |  | 0.008 |
| Blue VS Far Red | 30.49 | 688.29 | unknown | 0.44 |  | 0.002 |
| Blue VS Far Red | 25.04 | 686.79 | unknown | 0.16 |  | 0.004 |
| Red VS Far Red | 29.89 | 312.12 | unknown | 6.23 |  | 0.009 |
| Red VS Far Red | 25.92 | 449.11 | unknown | 2.48 |  | 0.002 |
| Red VS Far Red | 30.70 | 693.28 | unknown | 2.44 |  | 0.010 |
| Red VS Far Red | 32.03 | 677.28 | N713 | 2.05 | 4 | 0.005 |
| Red VS Far Red | 2.11 | 526.12 | unknown | 1.49 |  | 0.003 |
| Red VS Far Red | 32.48 | 819.27 | unknown | 0.18 |  | 0.003 |
| Blue VS Red | 28.98 | 481.13 | unknown | 4.08 |  | 0.007 |
| Blue VS Red | 19.24 | 433.11 | unknown | 3.85 |  | 0.002 |
| Blue VS Red | 29.71 | 429.14 | unknown | 3.76 |  | <0.001 |
| Blue VS Red | 21.34 | 333.06 | unknown | 2.96 |  | 0.005 |
| Blue VS Red | 28.96 | 855.25 | unknown | 2.55 |  | 0.001 |
| Blue VS Red | 14.57 | 351.07 | unknown | 2.34 |  | 0.009 |
| Blue VS Red | 4.81 | 345.08 | unknown | 2.21 |  | 0.003 |
| Blue VS Red | 22.04 | 449.11 | unknown | 1.91 |  | 0.004 |
| Blue VS Red | 13.42 | 595.17 | Naringenin-C-diglycoside | 1.83 | 2 | <0.001 |
| Blue VS Red | 28.10 | 515.12 | dicaffeoylquinic acid IV | 1.34 |  | 0.008 |
| Blue VS Red | 29.61 | 221.07 | unknown | 1.10 |  | 0.004 |
| Blue VS Red | 32.48 | 819.27 | unknown | 0.06 |  | 0.001 |
| Green VS White | 30.70 | 693.28 | unknown | 3.84 |  | 0.006 |
| Green VS White | 2.59 | 259.02 | unknown | 1.19 |  | <0.001 |
| Green VS White | 3.43 | 565.05 | unknown | 0.93 |  | 0.003 |
| Green VS White | 24.97 | 503.12 | unknown | 0.65 |  | 0.003 |
| Green VS White | 10.24 | 327.11 | unknown | 0.46 |  | 0.003 |
| Green VS White | 14.53 | 385.11 | Sinapic acid-hexose | 0.45 |  | 0.006 |
| Green VS White | 14.57 | 351.07 | unknown | 0.29 |  | 0.004 |
| Green VS White | 28.71 | 1152.54 | unknown | 0.29 |  | <0.001 |
| Green VS White | 15.34 | 351.07 | unknown | 0.26 |  | <0.001 |
| Green VS White | 16.53 | 351.07 | unknown | 0.23 |  | 0.009 |
| Green VS White | 22.73 | 463.09 | Quercetin 3-O-glucoside | 0.19 | 1 | 0.004 |
|  |  |  |  |  |  |  |


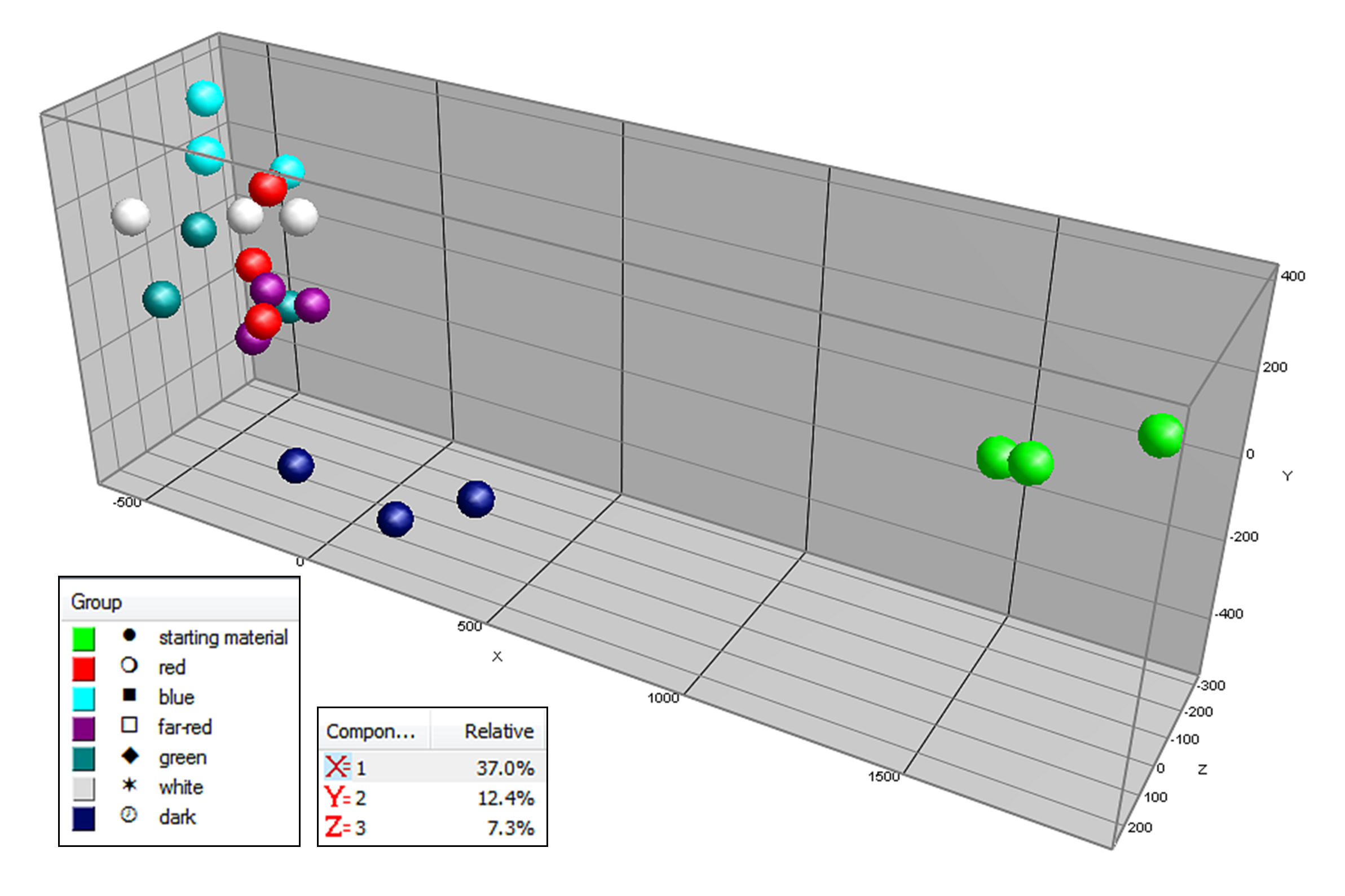


Supplemental Figure 1: Principle component analysis (PCA) of tomatoes treated with different light qualities, based on 437 tomato pericarp metabolites detected by LC-MS based untargeted metabolomics. The fruits were treated with different light spectrum treatments (350 μmol m^-2^ s^-1^) and darkness for 7 days (Exp. 2). PC1=X-axis=37% explained variation; PC2=Y-axis=12.4% explained variation; PC3=Z-axis=7.3% explained variation (Red treatment-red points, blue treatment-light blue points, far-red treatment-purple points, green treatment-dark green points, white treatment-white points, dark treatment-dark blue points, starting material-light green points).


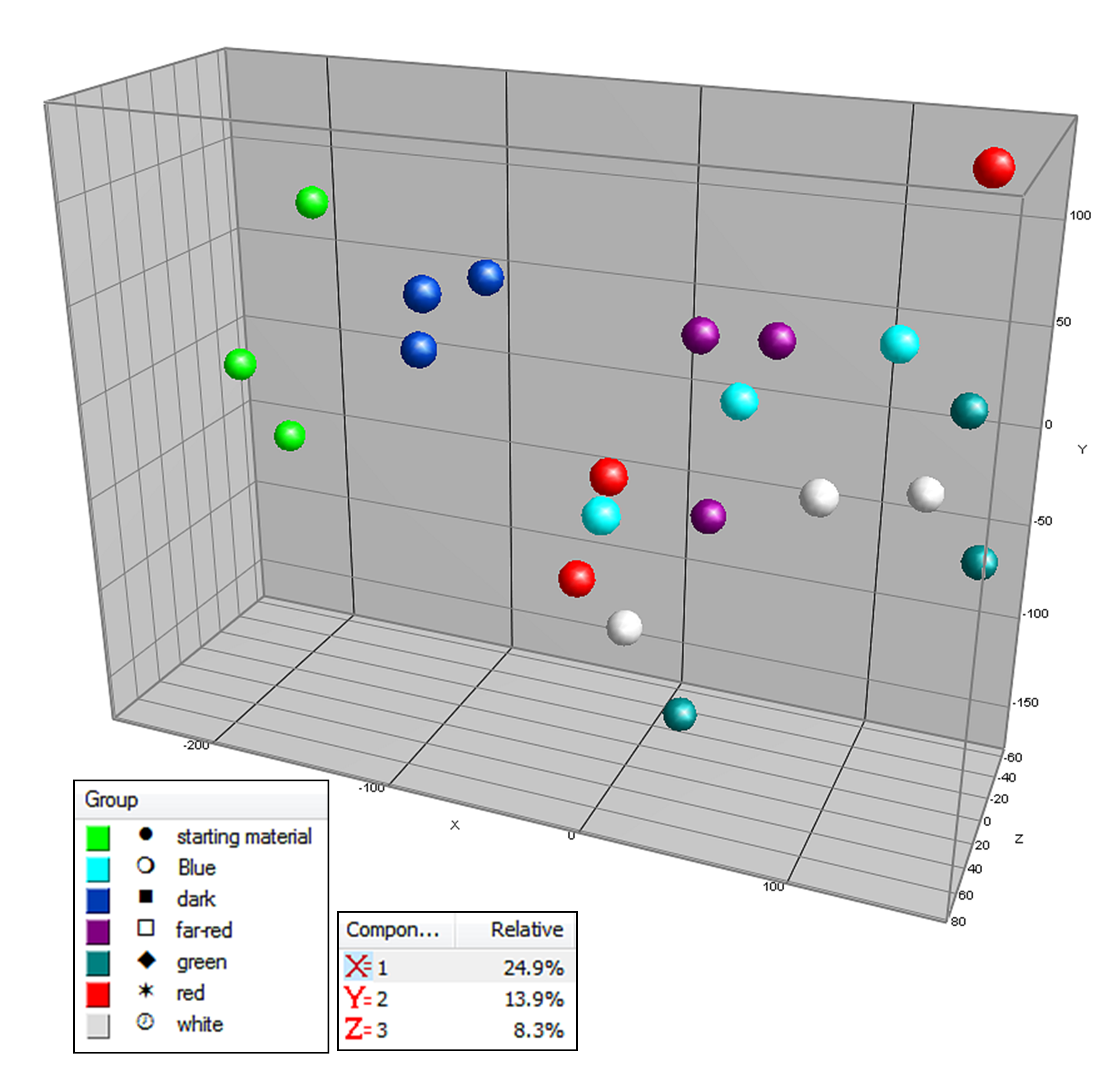


Supplemental Figure 2: Principle component analysis (PCA) of tomatoes treated with different light qualities, based on 109 tomato pericarp metabolites detected by GC-MS based untargeted metabolomics. The fruits were treated with different light spectrum treatments (350 μmol m^-2^ s^-1^) and darkness for 7 days (Exp. 2). PC1=X-axis=24.9% explained variation; PC2=Y-axis=13.9% explained variation; PC3=Z-axis=8.3% explained variation (Red treatment-red points, blue treatment-light blue points, far-red treatment-purple points, green treatment-dark green points, white treatment-white points, dark treatment-dark blue points, starting material-light green points).

Supplemental Figure 3: Metalign setting used for pre-processing the LCMS (A) and GCMS (B) raw data files. For explanation of the various parameters, see the Metalign manual at [www.metalign.nl](http://www.metalign.nl).

A.


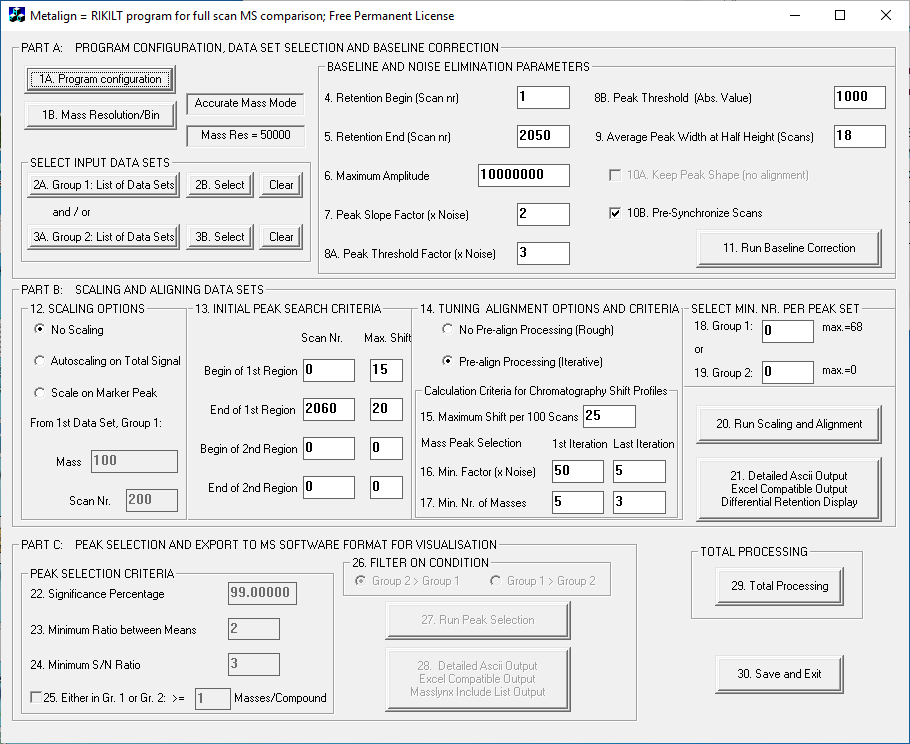


B.


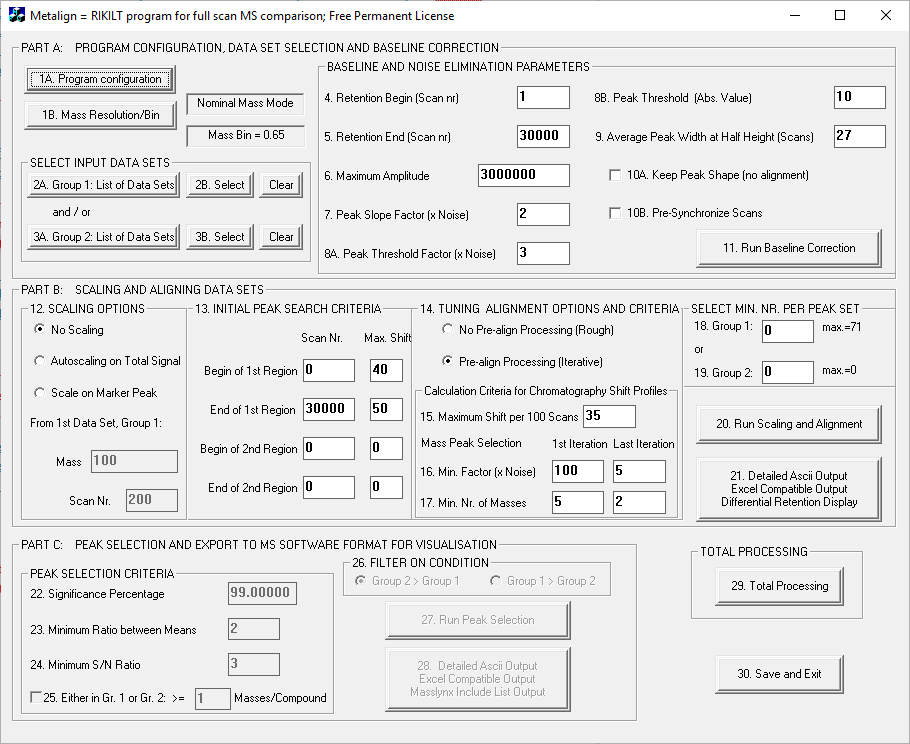

Supplement: Supplementary file 1 [file metabolites-10-00266-s001.zip › Ntagkas_etal_metabolomics_SupplementaryMaterial_Metabolites_V3_06062020.docx]
